# Quantitative evaluation of blinking in blepharospasm using electrooculogram-integrated smart eyeglasses

**DOI:** 10.1038/s41598-023-36094-4

**Published:** 2023-06-18

**Authors:** Ayano Yoshimura, Yuka Hosotani, Akiko Kimura, Hiroyuki Kanda, Youichi Okita, Yuji Uema, Fumi Gomi

**Affiliations:** Department of Ophthalmology, Hyogo Medical University, 1-1 Mukogawa-cho, Nishinomiya, Hyogo, 663-8501 Japan

**Keywords:** Eye abnormalities, Eyelid diseases

## Abstract

Smart eyeglasses with an integrated electrooculogram (EOG) device (JINS MEME ES_R^®^, JINS Inc.) were evaluated as a quantitative diagnostic tool for blepharospasm. Participants without blepharospasm (*n* = 21) and patients with blepharospasm (*n* = 19) undertook two voluntary blinking tests (light and fast) while wearing the smart eyeglasses. Vertical (*Vv*) and horizontal (*Vh*) components were extracted from time-series voltage waveforms recorded during 30 s of the blinking tests. Two parameters, the ratio between the maximum and minimum values in the power spectrum (peak-bottom ratio, Fourier transform analysis) and the mean amplitude of the EOG waveform (peak amplitude analysis) were calculated. The mean amplitude of *Vh* from light and fast blinking was significantly higher in the blepharospasm group than in the control group (*P*  < 0.05 and *P*  < 0.05). Similarly, the peak-bottom ratio of *Vv* from light and fast blinking was significantly lower in the blepharospasm group than in the control group (*P*  < 0.05 and* P*  < 0.05). The mean amplitude of *Vh* and peak-bottom ratio of *Vv* correlated with the scores determined using the Jankovic rating scale (*P*  < 0.05 and* P*  < 0.01). Therefore, these parameters are sufficiently accurate for objective blepharospasm classification and diagnosis.

## Introduction

Blepharospasm (BS) is an involuntary eyelid closure caused by excessive intermittent or sustained contraction of the orbicularis oculus muscle and is neurologically classified as a focal dystonia^1–3^. Symptoms of BS include excessive blinking, difficulty opening the eyelid, photophobia, droopy eyelids, foreign body sensation, eye pain, headache, and depression^4–6^. In severe cases, BS may result in functional blindness.

The etiology of BS is often unknown. BS-like symptoms can occur with certain neurologic or ophthalmic disorders. In particular, dry eye and BS often share symptoms, and more than 40% of BS patients have been diagnosed with dry eye^7–11^. Thus far, BS diagnosis is based on symptoms and drug history combined with blinking tests and other ophthalmological examinations. However, there are no established measurement tools and scales, and thus some cases can take several years for a definitive BS diagnosis.

The Jankovic rating scale (JRS), the first scale for eyelid spasm, classifies the severity and frequency of BS according to patients’ complaints^7^. This is currently the most widely used clinical scale, although it is a subjective and qualitative evaluation. Video recording systems have been incorporated to measure the blinking frequency and eyelid closure time to evaluate the efficacy of botulinum toxin treatment^12–14^. Osaki et al. estimated the upper eyelid energy power using a high-speed camera and micro light-emitting diodes^15,16^. Other tests for BS diagnosis include electromyography (EMG)^17^, and positron emission tomography (PET)^18–20^. However, these tests are not easy to conduct in routine clinical practice and cannot accurately assess the disease state.

We postulated that differences in the patterns of electrooculogram (EOG) between participants without BS and patients with BS could be used to diagnose and classify BS. Smart eyeglasses with an integrated EOG device are now commercially available. We used these smart eyeglasses to enable examinations under everyday situations. In this study, we analyzed the obtained EOG to identify and evaluate features distinguishing participants without BS from patients with BS for objective BS diagnosis.

## Methods

We conducted a prospective, comparative study at Hyogo Medical University Hospital (Japan) from March 2020 to July 2021. The research protocol followed the guidelines of the Declaration of Helsinki and was approved by the Institutional Review Board of Hyogo University of Medicine Hospital (Review Board Number 3389). All participants gave informed consent prior to participation in the study. Two groups were established: a group of patients with BS in the age range of 20 to 60 years and a control group of age- and sex-matched participants without blinking problems. BS diagnosis was made by two ophthalmologists on the basis of standard criteria in Japan^21^. For assessment of corneal epithelial fluorescein staining, we used NEI score^22^. Participants with a history of central nervous system disorders, hemifacial spasm, severe ptosis or skin laxity, ocular surface diseases (such as superior limbic keratoconjunctivitis), contact lens use, eyelid surgery, glaucoma medication-related eyelid changes, and other diseases or conditions that affect blinking were excluded. Participants with dry eye and assigned a score of 2 or more for superficial punctate keratopathy and/or a fluorescein break up time (BUT) of 3 s or less were also excluded. If participants used eye drops routinely, then they were allowed to continue with eye drops. In the BS group, patients who had been treated with botulinum toxin type A within 4 months were excluded. Participants with missing data or difficult-to-analyze data were also excluded.

All participants underwent ophthalmological examinations, including measurements with a slit lamp, measurements of the BUT, and evaluations of blinking. The severity and frequency of BS were determined using the JRS^7^ (0 = none, 1 = marked, 2 = mild, 3 = moderate, 4 = severe) for a total score (0–8). The control group had a score of 0. Then, participants underwent voluntary blinking tests while wearing the smart eyeglasses.

### Voluntary blinking tests

The blinking tests used in this study were based on the clinical guidelines for BS in Japan^21^. Participants wore smart eyeglasses in a sitting position with the chin and forehead on a fixed chin rest and were instructed to blink to the constant rhythm of a metronome. The rhythm was set to 80 beats per minute (bpm) for the light blinking test and 130 bpm for the fast blinking test, and measurements lasted at least 30 s. A video camera was set up at a distance of approximately 2 meters from the sitting position to record the blinks and monitor the blinking.

### Recording device

A commercial wearable device, namely smart eyeglasses with an integrated EOG device (JINS MEME ES_R^®^, JINS Inc.)^23^, was used to obtain voltage changes during voluntary blinking tests. Details of this device are available in a previous report^23^. The device comprises electrodes and a voltage sensor for EOG recordings. The voltage is measured unipolarly with three electrodes located at the bridge (center, C), left (L) nose pad, and right (R) nose pad.

The vertical (*Vv*) and horizontal (*Vh*) components can be obtained from the voltages continuously sampled at the three electrodes, C (*VC*), L (*VL*), and R (*VR*), using Eqs. (1) and (2)^23^:

1 $$Vv = VC - \left(VR+VL\right)/{2}$$

2 $$Vh=VR-VL$$

The voltage sensor has a sampling rate of 100 Hz and a measurement range of − 1500 μV to + 1500 μV. The recorded data are collected via wireless capabilities and stored in a personal computer in the comma separated value (CSV) file format.

### Waveform analysis

Components of time-series voltage waveforms, *Vv* and *Vh*, were analyzed using two different procedures: wave amplitude and Fourier transform. These analyses were conducted for three sections of the waveforms: the entire 30 s, initial 10 s, and last 10 s.

(i) Wave amplitude analysis

To obtain the wave amplitude, peaks were detected using the argrelmax function of the Scipy module in the Python3 environment. Among the detected peaks, peaks with amplitudes lower than the set threshold (40 μV) were excluded. The wave amplitude was defined as the distance from the minimum value of the waveform to the height of adjacent peaks. We calculated the wave amplitude of each peak and averaged the wave amplitudes in the waveform.

(b) Fourier transform analysis

To obtain frequency spectra, fast Fourier transform (FFT) of the waveforms was performed using the FFT function of the Numpy module in the Python3 environment. Because the rhythm was set to 80 bpm for the light blinking test, peaks were expected at 1.33 Hz and its harmonic frequencies in the frequency spectra of light blinking tests. Similarly, because the rhythm was set to 130 bpm for the fast blinking test, peaks were expected at 2.16 Hz and its harmonic frequencies in the frequency spectra of fast blinking tests. We focused on the fundamental, second harmonic, and third harmonic frequencies. The ratio of peak power to minimum power of each harmonic frequency band was calculated. We defined the average of the ratios of each spectrum as the peak-bottom ratio.

### Statistical analysis

Analyses were conducted using JMP Pro (version 15, SAS Institute Inc., Cary, NC). For each parameter (mean amplitude, peak-bottom ratio), the mean and standard deviation were calculated for both the control and BS groups. *T*-tests were used for normally distributed continuous variables.

Paired *t*-tests were used for correlation analysis of the amplitude and peak-bottom ratio. We evaluated correlations for the amplitude in four combinations: “*Vv* versus *Vh* in light blinking,” “*Vv* versus *Vh* in fast blinking,” “light blinking versus fast blinking in *Vv*,” and “light blinking versus fast blinking in *Vh*.” Moreover, we evaluated correlations for the peak-bottom ratio in the initial 10 s versus the last 10 s of the test.

In this study, the area under the curve (AUC) was used as an index to evaluate performance. Pearson’s correlation coefficients were calculated to evaluate the correlation between EOG parameters and JRS scores. A *P*-value < 0.05 was considered statistically significant.

## Results

### Characteristics of participants

The characteristics of 19 patients with BS (2 men and 17 women; mean age of 49.32 ± 11.80 years) and 21 participants without BS (6 men and 15 women; mean age of 45.29 ± 11.14 years) are shown in Table 1. In the BS group, 19 patients had BS and 17 patients were treated with botulinum toxin. Participants were assessed using the JRS (severity + frequency): 21 participants had a score of 0, 3 participants had a score of 2, 2 participants had a score of 3, 4 participants had a score of 4, 4 participants had a score of 5, 3 participants had a score of 6, 2 participants had a score of 7, and 1 participant had a score of 8.

**Table 1:** Characteristics of participants.

	Control group (*n* = 21)	BS group (*n* = 19)
Female, n (%)	15 (71.4)	17 (89.5)
Age, y (mean ± SD)	45.29 ± 11.14	49.32 ± 11.80
Jankovic Rating Scale, n (%)		
0	21 (100)	0
1	0	0
2	0	3 (15.8)
3	0	2 (10.5)
4	0	4 (21.1)
5	0	4 (21.1)
6	0	3 (15.8)
7	0	2 (10.5)
8	0	1 (5.3)

BS, blepharospasm; Data are expressed as the mean ± SD; Jankovic Rating Scale = Severity + Frequency.

### Features of waveforms captured by the EOG sensor

**Figure 1:**
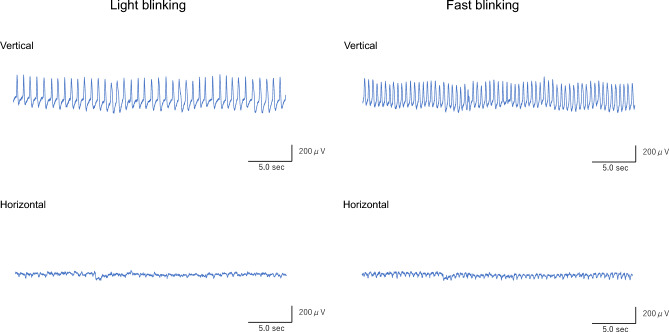
Representative EOG waveforms over the entire 30 s of blinking for the control group. Vertical waveforms show rhythmic peaks according to blinking, while horizontal waveforms are almost flat during both light and fast blinking tests. Abbreviations: EOG, electrooculogram; sec, seconds.

**Figure 2:**
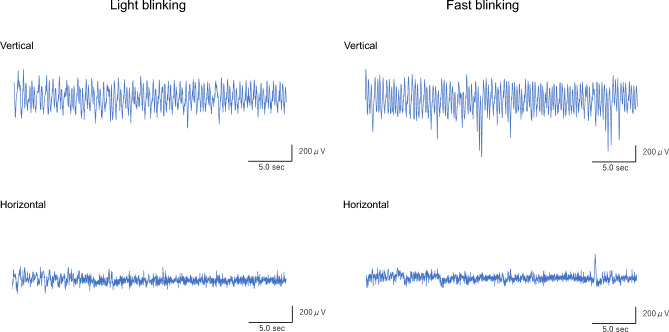
Representative EOG waveforms over the entire 30 s of blinking for the BS group. Vertical waveforms are varied and not rhythmic, and the horizontal waveforms of both light and fast blinking also fluctuate. Abbreviations: EOG, electrooculogram; BS, blepharospasm; sec, seconds.

Representative waveforms of light and fast blinking tests for the control and BS groups are shown in Figs. 1 and 2. In the control group, the vertical waveforms peaked according to blinking, while the horizontal waveforms were almost flat (Fig. 1). The amplitudes of the vertical waveforms varied in the BS group, while the amplitudes of the horizontal waveforms were higher in the BS group than in the control group (Fig. 2).

**Figure 3:**
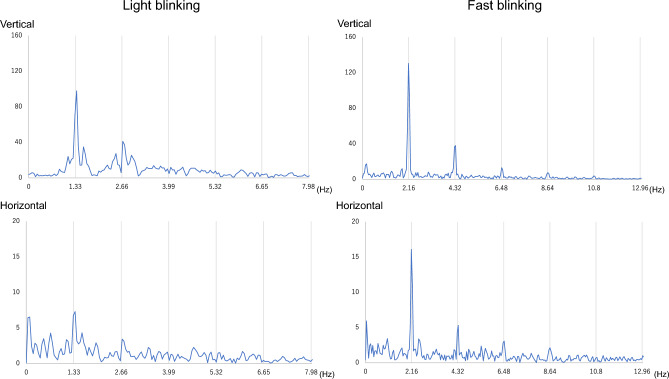
Fourier transform of representative EOG waveforms over the entire 30 s of blinking for the control group. The highest peaks of both vertical and horizontal waveforms of light and fast blinking occur at 1.33 Hz and 2.16 Hz, respectively. Abbreviations: EOG, electrooculogram.

**Figure 4:**
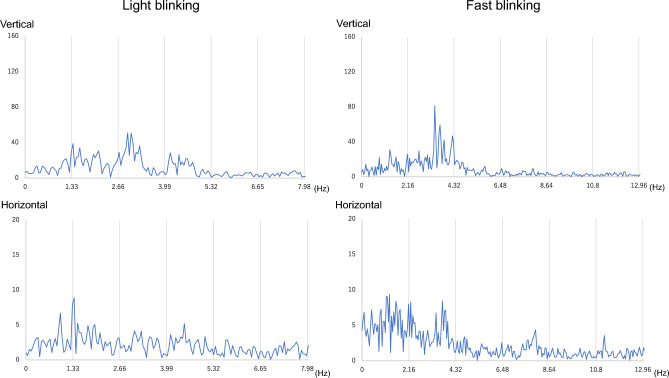
Fourier transform of representative EOG waveforms over the entire 30 s of blinking for the BS group. There are no constant peaks in all waveforms. Abbreviations: EOG, electrooculogram; BS, blepharospasm.

Power spectra of representative waveforms of light and fast blinking tests for the control and BS groups are shown in Figs. 3 and 4. The timing was set to 80 bpm for light blinking and 130 bpm for fast blinking, and thus peaks were expected every 1.33 Hz and 2.16 Hz, respectively. In the control group, both vertical and horizontal components had peaks associated with blinks (Fig. 3), whereas in the BS group, peaks were random (Fig. 4).

### Comparison of wave amplitudes

Differences in the wave amplitudes between the control and BS groups were analyzed according to the blinking period and are shown in Table 2. In *Vv*, the mean wave amplitude in the last 10 s of light blinking was significantly lower in the BS group than in the control group (*P* = 0.014). However, the mean wave amplitude in the initial 10 s and entire 30 s of light blinking did not differ significantly between the two groups. In *Vh*, the mean wave amplitudes over the entire 30 s of both light and fast blinking were significantly higher in the BS group than in the control group (*P* = 0.019 and* P* = 0.023, respectively). Moreover, the mean wave amplitudes in the initial 10 s and last 10 s of both light and fast blinking were significantly higher in the BS group than in the control group (*P* = 0.049, *P* = 0.034, *P* = 0.018, and* P* = 0.009, respectively).

**Table 2:** Comparison of amplitudes from representative EOGs between participants with and without BS.

		Blinking pattern	Control group (*n* = 21)	BS group (*n* = 19)	*P*-value*	AUC**
Mean amplitude (vertical components, µV)	Entire 30 s	Light blinking	384.76 ± 34.24	311.17 ± 35.99	0.147	0.624
		Fast blinking	421.38 ± 38.01	356.12 ± 39.96	0.244	0.612
	Initial 10 s	Light blinking	438.54 ± 35.88	345.31 ± 37.72	0.081	0.631
		Fast blinking	428.55 ± 40.03	349.25 ± 42.09	0.180	0.647
	Last 10 s	Light blinking	447.67 ± 35.06	316.42 ± 36.85	**0.014**	0.709
		Fast blinking	434.69 ± 34.37	339.95 ± 36.13	0.065	0.664
Mean amplitude (horizontal components, µV)	Entire 30 s	Light blinking	69.51 ± 24.01	154.68 ± 25.24	**0.019**	0.732
		Fast blinking	74.31 ± 3.49	155.36 ± 4.69	**0.023**	0.734
	Initial 10 s	Light blinking	91.91 ± 23.67	161.86 ± 24.89	**0.049**	0.679
		Fast blinking	88.94 ± 21.72	156.22 ± 22.84	**0.034**	0.709
	Last 10 s	Light blinking	80.99 ± 31.06	192.50 ± 32.65	**0.018**	0.720
		Fast blinking	71.94 ± 25.07	172.23 ± 26.36	**0.009**	0.762

*Statistical analysis with Student’s *t*-test.

**Area under the curve.

BS, blepharospasm; s, seconds; Data are expressed as the mean ± SD.

Significant are in value [bold].

The AUC of the mean amplitude in *Vh* was higher in the last 10 s than in the initial 10 s of both light and fast blinking (Table 2). In particular, the highest AUC (0.762) was obtained for the mean amplitude in *Vh* in the last 10 s of fast blinking.

For each participant, there were significant differences between the amplitudes of *Vv* and *Vh* over the entire period of light or fast blinking in the control (light blinking: *P* < 0.0001, fast blinking: *P* < 0.0001) and BS groups (light blinking: *P* = 0.0002, fast blinking: *P* < 0.0001). However, there were no significant differences between the amplitude of light and fast blinking for each participant in both the control (*Vv* : *P* = 0.440, *Vh* : *P* = 0.319, respectively) and BS groups (*Vv* : *P* = 0.125, *Vh* : *P* = 0.650, respectively).

### Comparison of peak-bottom ratios

Differences in the peak-bottom ratios between the control and BS groups were analyzed and are shown in Table 3. In *Vv*, the peak-bottom ratio over the entire 30 s of both light and fast blinking was significantly lower in the BS group than in the control group (*P* < 0.001 and* P* = 0.015, respectively). Moreover, the peak-bottom ratios in the initial 10 s and last 10 s of both light and fast blinking were significantly lower in the BS group than in the control group (*P* = 0.016, *P* = 0.024, *P* < 0.001, and* P* = 0.004, respectively). In *Vh*, the peak-bottom ratio in the last 10 s of light blinking was significantly lower in the BS group than in the control group (*P* = 0.004). The peak-bottom ratio in the first 10 s and the entire 30 s of light blinking did not differ significantly between the two groups.

**Table 3:** Comparison of peak-bottom ratios from Fourier transformed waveforms between participants with and without BS.

		Blinking pattern	Control group (*n* = 21)	BS group (*n* = 19)	*P*-value*	AUC**
Peak-bottom ratio (vertical components)	Entire 30 s	Light blinking	7.46 ± 0.64	3.62 ± 0.68	** < 0.001**	0.852
		Fast blinking	5.58 ± 0.61	3.33 ± 0.64	**0.015**	0.734
	Initial 10 s	Light blinking	6.33 ± 0.66	3.90 ± 0.70	**0.016**	0.739
		Fast blinking	4.23 ± 0.41	2.83 ± 0.43	**0.024**	0.721
	Last 10 s	Light blinking	6.96 ± 0.68	2.97 ± 0.71	** < 0.001**	0.882
		Fast blinking	4.73 ± 0.48	2.60 ± 0.50	**0.004**	0.749
Peak-bottom ratio (horizontal components)	Entire 30 s	Light blinking	1.63 ± 0.07	1.52 ± 0.07	0.277	0.594
		Fast blinking	3.06 ± 0.32	2.32 ± 0.33	0.114	0.707
	Initial 10 s	Light blinking	3.04 ± 0.38	2.46 ± 0.40	0.291	0.612
		Fast blinking	2.57 ± 0.29	2.08 ± 0.31	0.255	0.689
	Last 10 s	Light blinking	3.14 ± 0.26	1.99 ± 0.27	**0.004**	0.752
		Fast blinking	2.63 ± 0.25	2.07 ± 0.26	0.131	0.647

*Statistical analysis with Student’s *t*-test.

**Area under the curve.

BS, blepharospasm; s, seconds; Data are expressed as the mean ± SD.

Significant are in value [bold].

The AUC of the peak-bottom ratio in *Vv* was higher in light blinking than in fast blinking. In addition, the AUC of the peak-bottom ratio in *Vv* was higher in the last 10 s than in the initial 10 s of both light and fast blinking (Table 3).

In the control group, there were no significant differences between the peak-bottom ratios in the initial 10 s and last 10 s of both light and fast blinking, neither in *Vv* (light blinking: *P* = 0.164, fast blinking: *P* = 0.370, respectively) nor in *Vh* (light blinking: *P* = 0.602, fast blinking: *P* = 0.776, respectively). In the BS group, there were significant differences between the peak-bottom ratios in the initial 10 s and last 10 s of both light and fast blinking in *Vv* (light blinking: *P* = 0.023, fast blinking: *P* = 0.026, respectively) and of only fast blinking in *Vh* (*P* = 0.0095).

### Correlation between the JRS score and EOG parameters

Correlations between the JRS scores and EOG parameters for the control and BS groups were analyzed and are shown in Table 4. The mean amplitude of *Vh* and the peak-bottom ratio of *Vv* had significant positive correlations with the JRS scores.

**Table 4:** Correlation between EOG parameters and Jankovic rating scale score among all participants.

		Blinking pattern	r	*P*-value*
Mean amplitude (vertical components, µV)	Entire 30 s	Light blinking	− 0.208	0.197
		Fast blinking	− 0.163	0.316
	Initial 10 s	Light blinking	− 0.237	0.141
		Fast blinking	− 0.128	0.331
	Last 10 s	Light blinking	− 0.384	**0.014**
		Fast blinking	− 0.297	0.062
Mean amplitude (horizontal components, µV)	Entire 30 s	Light blinking	0.392	**0.012**
		Fast blinking	0.395	**0.012**
	Initial 10 s	Light blinking	0.349	**0.027**
		Fast blinking	0.409	**0.009**
	Last 10 s	Light blinking	0.374	**0.018**
		Fast blinking	0.462	**0.003**
Peak-bottom ratio (vertical components)	Entire 30 s	Light blinking	− 0.620	** < 0.0001**
		Fast blinking	− 0.431	**0.005**
	Initial 10 s	Light blinking	− 0.400	**0.010**
		Fast blinking	− 0.402	**0.010**
	Last 10 s	Light blinking	− 0.596	** < 0.001**
		Fast blinking	− 0.413	**0.008**
Peak-bottom ratio (horizontal components)	Entire 30 s	Light blinking	− 0.116	0.476
		Fast blinking	− 0.287	0.073
	Initial 10 s	Light blinking	− 0.229	0.155
		Fast blinking	− 0.254	0.114
	Last 10 s	Light blinking	− 0.499	**0.001**
		Fast blinking	− 0.212	0.189

*Statistical analysis with Pearson’s correlation coefficient.

EOG, electrooculogram; s, seconds.

Significant are in value [bold].

## Discussion

The purpose of this study was to investigate whether a wearable device equipped with an EOG device (JINS MEME ES_R^®^, JINS Inc.) could serve as a quantitative diagnostic tool for BS by providing differences in the characteristics of *Vv* and *Vh* between the control and BS groups. This wearable device was developed to acquire EOG in a simple and stress-free manner, with various applications currently being explored. A conventional EOG device uses a pair of electrodes placed at the inner and outer corner of the right and left eye, respectively, to measure horizontal eye movements as well as two electrodes placed above and below the eyelid cleft to measure vertical eye movements. The wearable device used in this study enables measurements in a natural state because it is equipped with a pair of electrodes on the left and right nose pads of the frame and electrodes on the bridge and nose pads of the frame to measure horizontal and vertical eye movements, respectively.

To the best of our knowledge, there has been no reported study using commercially available wearable devices to evaluate BS. Recent studies have used a variety of diagnostic tools and evaluation methods for BS, including high-speed video cameras to record eyelid movements^15,16^, neural network systems to evaluate facial expressions^14^, and soft nanomembrane sensors for EMG^24^. However, these methods are difficult to perform in routine clinical practice because of the complexity of the measurement methods and the invasive nature of equipment elements. We hypothesized that the integrated EOG device of the smart eyeglasses could easily provide quantitative differences in voltage fluctuations between the BS and control groups.

As expected, participants with BS could not blink to a set rhythm. The wave amplitudes of the vertical component showed huge variations among participants. The mean amplitude of *Vv* was significantly lower in the BS group than in the control group only in the last period of the blinking task. Generally, vertical voltage fluctuations during EOG measurements include EMG signals arising from periocular muscles associated with eye blinking^25,26^. When we checked the videos taken during voluntary blinking tests of patients, involuntary muscle contractions were observed and thus EMG signals derived from periocular muscles might affect *Vv* in the BS group, especially in the initial period of the blinking tasks. However, sustained eyelid closure with prolongation of the task can prevent patients from performing sufficient blinking movements, and thus in the later period, blinking might be less affected by periocular muscles than sustained eyelid closure and the amplitude of *Vv* is significantly lower in the BS group than in the control group.

Interestingly, the horizontal component also showed fluctuations in the BS group. The mean amplitude of *Vh* was significantly higher in the BS group than in the control group, and we believe that changes in the horizontal wave of the EOG can be used to distinguish between participants with and without BS, as shown in the relatively high AUC values. In particular, the highest AUC was obtained for the mean amplitude in *Vh* in the last 10 s of fast blinking, indicating that this parameter is accurate for objective assessment of BS.

Although the mean amplitude of *Vv* was significantly higher than that of *Vh* during light and fast blinking in both the control and BS groups, there was no significant difference in either *Vv* or *Vh* between light and fast blinking. These results suggest that there were individual differences in the potentials of each component.

Then, we focused on the peaks that appeared following FFT of the waveforms. The blinking tests were based on set rhythms, and thus in the control group, peaks coinciding with the rhythm appeared in both *Vh* and *Vv*. However, in the case of patients with BS, regular peaks did not appear owing to irregular blinking and contaminated noise, as shown in Fig. 4. The peak-bottom ratio of *Vv* was significantly lower in the BS group than in the control group, as shown in Table 3, and for each of the participants in the BS group, it was also significantly lower in the initial 10 s than in the last 10 s of the waveform. In contrast, the peak-bottom ratio of *Vh* was significantly lower in the BS group than in the control group only in the last 10 s of light blinking. Therefore, the peak-bottom ratio of *Vv* can be used to distinguish between participants with and without BS, as indicated by the higher AUC values.

The current study showed the amplitude of *Vh* and the peak-bottom ratio of *Vv* were useful to distinguish patients with BS from participants without BS, especially in later phase of the blinking task. This can be a significant characteristic of BS. Previously, Lueck et al. also pointed out a significant prolongation of saccade latency in the horizontal and downward gaze of patients with BS by capturing eye movements with a scleral magnetic search coil method^27^, although no analysis of the time course of blink loading has been reported so far. Moreover, the mean amplitude of *Vh* and peak-bottom ratio of *Vv* had significant positive correlations with disease severity as indicated by the JRS score. Therefore, these values are very important not only for diagnosis but for severity assessment.

The voluntary blinking test used in this study is based on tests documented in the clinical guidelines for BS in Japan^21^. They include three types of voluntary blinking tests, such as fast blinking for more than 10 s, light blinking, and strong blinking, and if participants cannot perform these tests, they are diagnosed as BS positive. Wakakura et al. used these blinking tests to assess the severity of BS, with a score from 0 to 3^8,28^. Although JRS is commonly used to classify the severity of BS and has been shown to be useful in the evaluation of botulinum toxin treatment^1,2,7,29,30^, the severity scale introduced by Wakakura et al. is based on blinking tests and thus the results presented might show a stronger correlation with these scores than with JRS scores^8,28^.

This study has several limitations. First, we did not examine any differences in blinking performance by age. In this study, the ages of participants were in the range of 20 to 60 years. Spontaneous blinking of participants in the age range of 40 to 89 years has been analyzed using the electromagnetic search coil method, revealing that the average number of blinks, average voltage value, and peak velocity decrease as the participant’s age increases from 60 years^31^. We found that older participants (60 to 90 years old) had difficulty blinking to the rhythm of a metronome when we checked the video recordings, and thus age-specific analysis of blinking will be necessary in the future. Second, we included participants with mild dry eye, and because participants with dry eye might exhibit blinking abnormalities, a comparative study of patients with and without concomitant dry eye will also be necessary. The effects of eye drops should be evaluated as well. Last, for patients with severe BS, it is difficult to capture the movement of the eyeballs using this device.

Despite these limitations, our study revealed that the wearable EOG device can provide quantitative data to differentiate patients with BS from participants without BS and to assess BS severity. Using quantitative data, we may assess changes in blinking before and after treatments, such as with botulinum toxin.

## Data Availability

The datasets used and/or analyzed during the current study are available from the corresponding author on reasonable request.
